# NF-κB: Governing Macrophages in Cancer

**DOI:** 10.3390/genes15020197

**Published:** 2024-01-31

**Authors:** Jessica Cornice, Daniela Verzella, Paola Arboretto, Davide Vecchiotti, Daria Capece, Francesca Zazzeroni, Guido Franzoso

**Affiliations:** 1 Department of Immunology and Inflammation, Imperial College London, London W12 0NN, UK; j.cornice@imperial.ac.uk (J.C.); p.arboretto@imperial.ac.uk (P.A.); 2 Department of Biotechnological and Applied Clinical Sciences (DISCAB), University of L’Aquila, 67100 L’Aquila, Italy; davide.vecchiotti@univaq.it (D.V.); daria.capece@univaq.it (D.C.); francesca.zazzeroni@univaq.it (F.Z.)

**Keywords:** NF-κB, tumor-associated macrophages (TAMs), tumor microenvironment (TME)

## Abstract

Tumor-associated macrophages (TAMs) are the major component of the tumor microenvironment (TME), where they sustain tumor progression and or-tumor immunity. Due to their plasticity, macrophages can exhibit anti- or pro-tumor functions through the expression of different gene sets leading to distinct macrophage phenotypes: M1-like or pro-inflammatory and M2-like or anti-inflammatory. NF-κB transcription factors are central regulators of TAMs in cancers, where they often drive macrophage polarization toward an M2-like phenotype. Therefore, the NF-κB pathway is an attractive therapeutic target for cancer immunotherapy in a wide range of human tumors. Hence, targeting NF-κB pathway in the myeloid compartment is a potential clinical strategy to overcome microenvironment-induced immunosuppression and increase anti-tumor immunity. In this review, we discuss the role of NF-κB as a key driver of macrophage functions in tumors as well as the principal strategies to overcome tumor immunosuppression by targeting the NF-κB pathway.

## 1. Introduction

Macrophages represent one of the major lines of host defense in the innate immune system, able to kill pathogens and induce inflammatory response [1]. They control tissue repair and homeostasis via the extracellular matrix remodeling and scavenging of cellular debris and apoptotic cells [2]. Macrophages are highly versatile and can exert several different functions by changing their transcriptional profile based on the anatomic location and physiologic or pathophysiologic conditions [1,3].

In the tumor microenvironment (TME), the complex interactions between tumor cells, immune cells, endothelial cells, fibroblasts, and the extracellular matrix shapes tumor clinical behavior through the release and uptake of several angiogenic, mitogenic, immunosuppressive, or pro-migratory factors that may either inhibit or stimulate tumor progression [4]. Chemoattractants, produced by malignant cells and the stromal tumor compartment, such as C-C Motif Chemokine Ligand 2 (CCL2), vascular endothelial growth factor (VEGF), CXCL12 (SDF1) and colony-stimulating factor-1 (CSF-1), recruit monocytes from the bloodstream that migrate into tumor site [5,6]. Different studies showed that the tissue-resident macrophages (M_TR_) (e.g., microglia, Kupffer cells, alveolar macrophages) are responsible for regulating tissues homeostasis and inflammation, and during tumorigenesis, they developed progressively into pro-tumoral phenotype within the TME in presence of various molecules such as interleukin-10 (IL-10) [7,8,9]. Macrophages recruited in the tumor site, called tumor-associated macrophages (TAMs), are the major population of leukocytes in the TME, and display a high phenotype plasticity [10]. TAMs acquire a distinct phenotype and activation status and can exert anti- or pro-tumor activities through the expression of different functional programs [11]. 

There is a common agreement that macrophages can polarize into two different subtypes, the so-called M1-like and M2-like macrophages according to different stimuli. The M1 macrophages are “classically” activated by microbial products or soluble cytokines (e.g., lipopolysaccharide (LPS), tumor necrosis factor-α (TNF-α), interleukin-1β (IL-1β), and interferon-γ (IFN-γ) produced by activated CD4^+^ T helper (Th) 1 cells, CD8^+^ T cytotoxic cells, and natural killer (NK) cells, and show specific surface markers such as toll-like receptor(TLR)-2 (TLR-2), TLR-4, CD80, CD86, inducible nitric oxide synthase (iNOS), and major histocompatibility complex-II (MHC-II) [12]. M1 macrophages secrete nitric oxide (NO), reactive oxygen species (ROS) and various cytokines and chemokines (e.g., TNF-α, interleukin(IL)-1α (IL-1α), IL-1β, IL-6, IL-12, C-X-C motif chemokine ligand (CXCL) 9 (CXCL9), and CXCL10) which trigger the activity of NK and cytotoxic T cells [13]. In addition, these secreted factors activate unpolarized macrophages promoting the M1 state, in a positive feedback loop [14]. 

The M2 macrophages are “alternatively” activated by anti-inflammatory molecules such as glucocorticoid hormones and Th2 cytokines (interleukin (IL)-4 (IL–4), IL-10, and IL-13), as well as apoptotic cells and immune complexes [15]. As for M1, specific surface markers such as CD206, CD163, CD209, mannitol receptor, Ym1/2 and FIZZ1 characterize M2 macrophages. They also express anti-inflammatory cytokines such as IL-10, transforming growth factor-β (TGF-β), and chemokines (e.g., chemokine (C-C motif) Ligand(CCL) 1 (CCL1), CCL17, CCL22, CCL24) that contribute to dampen the inflammatory response and maintain macrophages into M2 phenotype by acting autocrinally [16,17]. 

Given the high plasticity of macrophages, the M2 phenotype has been subdivided into M2a, M2b, M2c and M2d subtypes, that differ both for stimuli and exerted functions. M2a macrophages, activated by IL-4 and IL-13, express higher levels of IL-10, TGF-β, CCL17 and CCL22 and promote cell growth, tissue repair and endocytic activity. Immune complexes, TLR ligands and interleukin-1 receptor (IL-1R) agonists activate M2b macrophages that regulate the intensity of inflammatory response and immune reaction via releasing TNF-α, IL-1β, IL-6 and IL-10. M2c macrophages, induced by IL-10, suppress immune responses and are responsible for tissue remodeling. Finally, M2d macrophages release IL-10 and VEGF and thus promote tumor progression and angiogenesis [10,17,18,19,20].

Recently the transcriptomic and proteomic analyzes of TAMs have identified different macrophage subpopulations in the TME, that go beyond the simple dichotomy “M1-M2” system, highlighting the presence of a more complex population of macrophages, highly plastic and heterogeneous [13,14]. Hence, several pieces of evidence indicated that microRNAs (miRNAs) [21], non-coding RNAs [22], extracellular vesicles (EVs) [23,24], and epigenetic modification [25] contribute to shape TAM phenotype in the TME, suggesting that the M1/M2 model has numerous limitations [13]. Although the current classification of macrophages is challenging as several heterogeneous subsets have been identified within the TME, here we use the common M1 and M2 classification to explain the role of NF-κB in TAM polarization. 

TAMs respond to the local signals provided by TME depending on tumor type and stage [26,27,28]. Although in the TME M2 macrophages are the most abundant population, M1 macrophages can be present in the TME during pathological conditions [29,30]. 

The balance between M1/M2 phenotypes and the switch between these two extreme borders of macrophage polarization is finely regulated by an intricate network of receptors and signaling pathways (e.g., JAK/STATs, MAPK, PI3K/AKT, NOTCH and NF-κB) [31,32]. 

Among these crucial signaling pathways, NF-κB is a key regulator of macrophage function in cancers, tipping the balance between the immunosuppressive, pro-tumoral activity and the pro-inflammatory, protective functions of TAMs [15]. Given the central role during tumorigenesis, TAMs represent a potential target for cancer treatment. NF-κB family of transcription factors plays a critical role in most physiological and pathological processes such as cell proliferation, survival, apoptosis [33,34], inflammation [35], immune response [36,37] tumor progression, invasion, metastasis, and angiogenesis [38]. NF-κB is also responsible for the activation and differentiation of innate immune cells and T cells [39] and the regulation of macrophage gene expression patterns [15].

The NF-κB family is composed of five structurally related DNA-binding subunits, consisting of the homo- and heterodimers of p50, p52, c-Rel, RelA (p65), RelB and c-Rel, which control transcription of target genes by binding specific DNA elements, called κB enhancers [40,41,42]. In resting cells, the NF-κB complexes are retained into the cytoplasm, bound to the inhibitory proteins of IκB family [43]. A wide range of stimuli, including microbial and viral infection products, stress, pro-inflammatory cytokines, and antigen receptors can trigger NF-κB activation [44] leading to the phosphorylation of IκBs by the IκB kinase (IKK) complex. In turn, IκBs trigger the polyubiquitination and proteolysis of the IκB inhibitors, leading to the translocation of the NF-κB complexes in the nucleus, where they drive the transcription of several target genes [45]. Based on stimuli, NF-κB activation involves different signaling pathways including the canonical, the non-canonical and the atypical pathways [39]. 

The canonical pathway is activated by microbial products, IL-1β, damage-associated molecular patterns (DAMPs), pattern-recognition receptors (PRRs), T-cell receptor (TCR) and B-cell receptor (BCR) [46,47] and leads to the phosphorylation of the IκB-family members (IκBα, IκBβ and IκBɛ) by the IκB kinase (IKK), complex, which is composed of two catalytic subunits, IKKα and IKKβ, and a regulatory subunit, IKKγ (also named NF-κB essential modulator, or NEMO). The IκB kinase IKKβ phosphorylates the IκB inhibitor molecules via an IKKγ/NEMO mechanism leading to their proteasomal degradation, via a ubiquitin-proteasome system. The active NF-κB dimers, predominantly the heterodimer p50/p65, translocate to the nucleus and induce the expression of several target genes [40,48]. 

The non-canonical pathway responds to different stimuli, such as LTβR, BAFFR, CD40 (belonging to TNFR superfamily members) and RANK, and activates a different response signal. The non-canonical pathway relies on NF-κB-inducing kinase (NIK) for the phosphorylation of IKKα and the subsequent processing of the NF-κB2 precursor protein (p100) that leads to the release of mature NF-κB2/p52-RelB heterodimers. The NF-κB2/p52-RelB complex translocates to the nucleus and regulates the transcription of non-canonical NF-κB target genes [49].

The atypical NF-κB activation pathway is triggered by factors involved in the ageing process, such as endoplasmic stress response, oxidative stress, mitochondrial dysfunction, and DNA damage. In the atypical pathway, NF-κB induces the transcription of pro-survival genes and activates genes responsible of ROS scavenging and inhibition of calcium release from the ER, to protect organelles from stress [50]. 

In this review, we will discuss the role of NF-κB in TAMs and how targeting the NF-κB pathway could be a promising approach to overcome tumor immunosuppression.

## 2. The NF-κB Pathway in TAMs 

**Figure 1:**
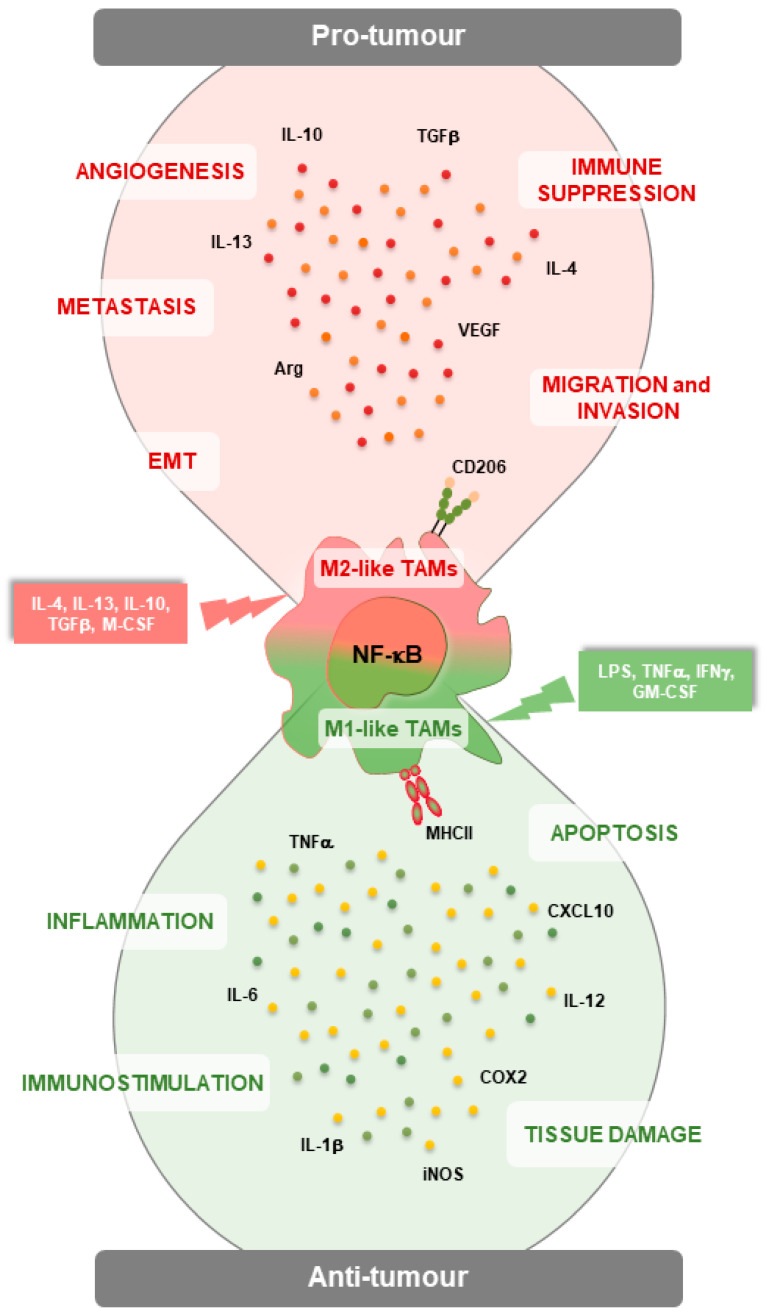
NF-κB signaling in M1- and M2-like tumor-associated macrophages (TAMs) in the tumor microenvironment (TME). NF-κB activation can polarize myeloid cells towards M1-like macrophages, which counteract tumorigenesis by promoting inflammation, immunostimulation, tissue damage, and apoptosis of cancer cells by inducing several molecules such as TNF-α, IL-12, iNOS, COX2 and IL-6. By contrast, NF-κB activation can shift macrophages towards M2-like anti-inflammatory TAMs, which promote tumor growth, angiogenesis, metastasis, and EMT, as well as the establishment of an immunosuppressive TME.

NF-κB plays a key role in TAM polarization during tumorigenesis (Figure 1) [15,38]. Accordingly, NF-κB, in response to activating stimuli such as TLR ligands, IL-1β, and TNF-α, can directly regulate the transition of macrophages toward M1 phenotype, usually exerting a tumor suppressor function, while, in different contexts, NF-κB activation can induce the transcription of many genes responsible for M2 polarization, thus promoting tumor growth (Table 1) [14,51,52,53].

Weigert and collaborators demonstrated that sphingosine-1-phosphate (S1P) produced by apoptotic tumor cells suppresses TNF-α production and increases interleukin-8 (IL-8) and IL-10 levels, thus promoting macrophage polarization toward an alternative activated (M2) phenotype in vitro. They showed that in response to LPS, S1P and apoptotic cancer cells inhibit the activation of NF-κB in macrophages. Accordingly, the reduced levels of S1P after genetic inhibition of sphingosine kinase 2 (Sphk2), restored M1 macrophages in vitro [54,55,56]. Recently, Shan and colleagues demonstrated that mechanical stretch (MS) (e.g., Flexcell Tension system) promotes M1 macrophage phenotype in an NF-κB-dependent manner, thus increasing tumoricidal effects in vitro and reducing tumor growth in vivo. The authors demonstrated that macrophages stretched with the Flexcell Tension system increase the levels of M1-related genes such as iNOS, TNF-α, IL-1β, and IL-6, as well as the release of M1 cytokines. The MS induces the up-regulation of focal adhesion kinase (FAK) that, in turn, activates NF-κB signaling, thus promoting the transcription of genes responsible of the M1 phenotype activity. Accordingly, NF-κB inhibition reduces the expression of M1 target genes in vitro [57]. In vivo study demonstrated that an intratumoral injection of macrophages treated with MS enhances M1 macrophage polarization within the TME and increases apoptosis of cancer cells, thus reducing melanoma growth [58]. 

In accordance with the role of MS to promote M1 polarization, Gao and collaborators demonstrated that tumor necrosis factor–related apoptosis-inducing ligand (TRAIL) plays an important role in re-educating macrophages towards an antitumor phenotype by inducing the activation of NF-κB as well as the expression of pro-inflammatory cytokines such as IL-1β, IL-6 and TNF-α that in turn promote a cytotoxic effect in the tumor cells [59]. The authors showed that TRAIL also enhanced the expression of miR-146a via NF-κB and its overexpression blocked the production of pro-inflammatory cytokines suggesting that miR146a negatively controls the immunosuppressive phenotype. The modulation of this immune response regulated by the TRAIL/NF-κB/miR-146a axis identified TRAIL as a potential target to re-educate macrophages in tumor tissues. 

Studies conducted by Lee and collaborators pointed out how NF-κB activation plays an important role in controlling the communication between tumor cells and TAMs through TLR4, reporting how NF-κB activation, on one hand, sustains cancer cell proliferation and invasion, and on the other hand, induces TAMs to release inflammatory cytokines and angiogenic factors in the TME, that in turn, support tumor proliferation, thus creating a vicious circle. They demonstrated that TLR4 signaling is the mediator of the NF-κB activation in TAMs. Consistently, TLR4 deficient TAMs showed a decreased NF-κB activity, and a reduced production of inflammatory and angiogenic factors, thus limiting tumor growth in vivo. Furthermore, TLR4 KO TAMs were not able to induce the activation of NF-κB in tumor cells. In contrast, macrophages TLR4 wild-type adoptive transferred in TLR4-deficient mice bearing tumor, showed a significantly higher NF-κB activity, enhanced release of inflammatory factors such as TNF-α and VEGF, thus prompting an increased NF-κB activity in tumor cells and tumor growth in vivo [60]. Therefore, targeting TLR4 in TAMs could be an attractive therapeutic strategy to counteract tumor growth in cancer patients. 

It is recognized that NF-κB can exert both anti-tumor and pro-tumor functions within the TME (Figure 1). In fact, the NF-κB activation can either promote the polarization of macrophages toward a pro-inflammatory anti-tumor phenotype or, at the same time, sustain the immunosuppressive activity of other cells such as Treg, macrophages, and dendritic cells, leading to tumor growth [61]. Yang and collaborators demonstrated that M-CSF stimulation increased the expression of c-Jun, a member of the AP-1 family, which in turn induces the macrophage polarization toward the M2 phenotype. Additionally, they showed that NF-κB synergizes with c-Jun to promote macrophage transformation from M1 to M2. Immunoprecipitation experiments confirmed the interaction between c-Jun and p50 after M-CSF stimulation, interaction that weakened in absence of macrophage colony-stimulating factor (M-CSF) or after treatment with a p50 inhibitor, andrographolide [62]. 

Although the activation of NF-κB is important for inducing the M2 phenotype, isolated TAMs from several well-established tumors have reduced NF-κB [15,63,64]. Saccani and collaborators demonstrated that high expression of the p50 NF-κB inhibitory homodimer inhibits M1 activation of TAMs and fosters tumor progression. Accordingly, TAMs isolated from mice knockout for p50 showed normal M1 activation with secretion of inflammatory cytokines and reduced tumor growth [65]. 

Kühnemuth and Michl demonstrated that the Cut-like homeobox 1 (CUX1), a homeodomain transcription factor expressed in different tumor types, acts as an antagonist of NF-κB signaling in TAMs. CUX1, which is the transcriptional target of the immunosuppressive cytokine TGFβ, exerts its action by displacing RelA from the promoters of several genes (e.g., CXCL10, CCL5) related to the M1-phenotype and by mediating the deacetylation of RelA through the recruitment of histone deacetylase 1 (HDAC1) to the promoters of NF-κB target genes. Furthermore, CUX1 inhibits the secretion of pro-inflammatory factors and supports tumorigenesis [66]. The inactivation of NF-κB by CUX1 inhibits the transactivation of inflammatory cytokines regulated by this transcription factor in established tumors [66]. Another mechanism that inhibits TAM anti-tumor activities is the degradation of NF-κB via selective autophagy. In vitro studies demonstrated that TLR2 signaling induces the accumulation of ubiquitinated NF-κB p65, that in turn forms aggresome-like structures (ALS) in the cytoplasm of M2 but not in M1 polarized macrophages. These structures are then recognized by the ubiquitin-binding proteins p62/SQSTM1 (sequestosome 1) and degraded via lysosomes. In addition, the authors showed that autophagy-dependent NF-κB p65 degradation is supported by sustained ERK1/2 phosphorylation that is triggered by TLR signaling [67]. 

NF-κB is also involved in the sophisticated mechanisms that regulate TAMs’ action in the processes of cancer cell invasion and metastasis [68]. A characteristic feature of the TME is the crosstalk between pericytes (PCs), cancer-associated fibroblasts (CAFs) and TAMs, that together coordinate the molecular mechanisms responsible of metastasis [68]. TAMs play important roles in promoting cancer cell dissemination [69]. Accordingly, interleukin-33 (IL-33) produced by CAFs drives the release of Th2-associated cytokines that polarize macrophages toward the M2 phenotype. IL-33-stimulated TAMs show an increase in NF-κB-mediated Matrix metalloproteinase-9 (MMP9) expression, that in turn degrades the extracellular matrix protein laminin and allows the extravasation and dissemination of tumor cells, suggesting that the IL-33-NF-κB-MMP9-laminin axis moderates the CAF-TAM crosstalk to foster cancer metastasis [68]. 

It Is well known that TME is characterized by low levels of oxygen (hypoxia) which promotes tumor progression and resistance to therapy. In addition, hypoxia enhances macrophage recruitment, thus conferring aggressiveness [70,71]. In this scenario, tumor cells and macrophages activate pro-angiogenic programs mediated by NF-κB-regulated hypoxia inducible factor 1 (HIF-1) that promote tumor cell adaptation and proliferation as well as TAM recruitment and oncogenic activities [72,73]. 

Studies showed that TAMs, together with other immune cells (e.g., myeloid-derived suppressor cells (MDSCs), T-regulatory cells (Treg)), infiltrate the hypoxic regions within the tumor and inhibit their anti-tumor function [71,74]. A study conducted by Delprat and colleagues indicated that cycling hypoxia (cyH), also called intermittent hypoxia, promotes the M1-like phenotype of macrophages via activation of JNK/p65 signaling pathway [75]. In this study the authors demonstrated that cyH promotes and amplifies a pro-inflammatory phenotype in non-activated (M0) and M1 macrophages by increasing the expression of M1 markers such as TNF-α, IL-8, CXCL10, Macrophage inflammatory protein 2 (MIP-2). This pro-inflammatory phenotype in human M0 and M1 macrophages was due to an increased activation of c-jun/NF-κB signaling. Accordingly, p65 and JNK ablation inhibits the pro-inflammatory phenotype induced by cyH, suggesting that c-jun-p65 axis regulates the cyH-mediated M1 macrophages [75]. 

TAMs support tumor resistance by regulating drug metabolism and/or secreting cytokines such as IL-6 in several cancer types. Additionally, M2-TAMs promote angiogenesis and tumor relapse [76]. A recent work showed that NF-κB is involved in the development of chemo and radiotherapy resistance, as well as in tumor response to therapy. In particular, several chemotherapeutic agents, such as taxol, cyclophosphamide, and cisplatin induce the up-regulation of proinflammatory cytokines such as TNF-α, IL-12, INOS, cyclooxygenase-2 (COX2), and the downregulation of anti-inflammatory factors like IL-10 and TGFβ via NF-κB activation. In addition, cisplatin and carboplatin treatments enhance the activation of the NF-κB pathway through the chemotherapy-induced DNA damage response (DDR), thus sustaining the polarization of monocytes toward M2-like macrophages in the TME [77]. Although radiotherapy affects TAM recruitment and phenotype in cancer, its role in reprogramming TAMs towards an anti-tumor phenotype remains unclear [78]. It is known that NF-κB plays an opposite role in TAM response during radiotherapy depending on the dose irradiation. Indeed, low radiation doses polarize macrophages towards a pro-tumoral phenotype by reducing the expression of IL-1β through the increase in the nuclear translocation of p50-p50 homodimer and the inhibition of p65 translocation [79]. On the contrary, moderate doses of radiations reprogram macrophages into M1 phenotype by inducing higher p65-p50 transcriptional activity, which in turn results in increased TNF-α, IL-6 and IL-8 production [80]. The immunosuppressive phenotype is maintained at high irradiation doses, where sustained activation of p50 keeps TAMs in an M2 polarization state (Table 1) [81].

**Table 1:** The NF-κB pathway in TAMs.

Tumor/Cell Type	Stimuli	NF-κB Pathway Component	TAMs Phenotype	Effect	Ref.
Breast carcinoma	SP1	p65/p50	M2	Pro-tumor	[54,55,56]
Melanoma	Mechanical stretch	p65	M1	Anti-tumor	[57,58]
Lung cancer	TRAIL	p65	M1	Anti-tumor	[59]
Melanoma, Bladder cancer	HSPs/TLR4	NF-κB	M2	Pro-tumor	[60]
Breast cancer	M-CSF	p50	M2	Pro-tumor	[62]
Pancreatic cancer	CUX1	p65 (RelA)	M2	Pro-tumor	[66]
Hepatoma	TLR2	p65	M2	Pro-tumor (Autophagy)	[67]
Pancreatic cancer	IL-33	IκBα	M2	Pro-tumor (Dissemination/ Metastasis)	[68,69]
Hepatocellular carcinoma	Hypoxia	IKKβ	M2	Pro-tumor (Angiogenesis, EMT)	[71]
hTHP1/mBMDM (Human monocytic cell line/ murine bone marrow-derived macrophage)	Cycling hypoxia	p65	M1	Anti-tumor (M0-M1 transition)	[75]
Ovarian cancer	Chemotherapeutic agents	IKK	M2	Pro-tumor	[77]
THP1 (monocytic cell line)	Low radiation doses	p50/p50; p65	M2	Pro-tumor (Reduced M1 cytokines)	[79]
THP1 (monocytic cell line)	Moderate radiation doses	p65/p50	M1	Anti-tumor	[80]
Mammary Carcinoma, Pancreatic adenocarcinoma	High radiation doses	p50	M2	Pro-tumor	[81]

## 3. NF-κB Signaling in TAMs: The Lesson from Different Human Cancer Types

### 3.1. Hepatocellular Carcinoma

According to the Global Cancer Statistics 2020, hepatocellular carcinoma (HCC) is the third leading cause of cancer death and the sixth most common cancer worldwide [82]. Many patients cannot benefit from the most common effective treatments, such as surgical resection, local tumor ablation, and liver transplant due to tumor size or the invasion and spreading of cancerous cells in other tissues and sites [83]. TME plays an important role in HCC progression and TAMs can mediate the acquisition and maintenance of tumor cell stemness conferring chemo and radiotherapy tolerance and resistance [84]. It is known that high density of TAMs in HCC has been associated to unfavorable prognosis [85]. This relationship is due to the mutual influence that tumor cells and TAMs exert on each other. In fact, the release of several factors such as M-CSF, CCL2, VEGF, and TGF-β and the expression of surface markers (e.g., glypican-3) on cancer cells are responsible of TAM recruitment and polarization. On the other hand, recruited and activated TAMs in HCC release many cytokines, chemokines and growth factors that are responsible for tumor cell proliferation, angiogenesis, extravasation, invasion and metastasis, as well as the suppression of anti-tumor immune response [72]. The NF-κB pathway plays a fundamental role in the establishment of HCC. It has been demonstrated that it exerts opposite functions based on the stage and development of the disease as well as the cell type where NF-κB is activated. NF-κB activation showed a tumor suppressor function in cancerous epithelial and parenchymal cells, while explaining a tumor promoting function if activated in Kupffer cells, the resident macrophages of the liver. In this scenario, the inflammatory cytokines released by activated Kupffer cells provide a pro-survival and proliferative stimulus to malignant hepatocytes mediated by myeloid-differentiation-factor-88 (MyD-88)/NF-κB pathway, thus sustaining tumorigenesis. The inhibition of NF-κB signaling in macrophages but not in hepatocytes reduces the production of pro-inflammatory cytokines and tumor growth [86,87,88,89].

A comparative analysis of RNA sequencing and whole-genome expression profiling of TAMs and M0 macrophages in HCC identified the upregulation in TAMs of the S100 calcium-binding protein A9 (S100A9) gene, which has a potential impact on HCC prognosis. In fact, the expression of S100A9 is correlated with poor clinical outcomes in patients with HCC [90]. It has been found that the S100A9 stimulates the NF-κB-dependent secretion of CCL2, the major chemoattractant for TAMs, thus promoting the recruitment, infiltration, and activation of TAMs. Furthermore, S100A9 secreted by TAMs induces a strong NF-κB activation and an increased expression of stemness-associated genes in HCC cells in a dose-dependent manner, resulting in an enhanced sphere formation ability and self-renewal in vitro. The knockdown of advanced glycosylation end product-specific receptor (AGER), the receptor of S100A9, eradicates the NF-κB-mediated pro-tumorigenic effects of S100A9, suggesting that S100A9 controls the crosstalk between tumor cells and TAMs and could be a potential target for treating HCC patients [90,91].

The oxidored-nitro domain-containing protein1 (NOR1) is overexpressed in human HCC tissues and correlates with advanced clinical stage and poor prognosis [92]. In vivo study demonstrated that in diethylnitrosamine (DEN)-induced HCC, NOR1 is overexpressed in F4/80 positive macrophages and decreases M1-like markers (e.g., iNOS) and increases M2-like markers, such as arginase-1 (Arg1) and IL-10, thus promoting M2 polarization. Consistently, loss of NOR1 ablates NF-κB p65 expression, impairs the production of inflammatory cytokines such as IL-6 and TNF-α in mice and reduces DEN-induced HCC, suggesting that NOR1/NF-κB plays a role in TAMs polarization and HCC development [92].

Another protein involved in promoting tumorigenesis and M2-like polarization is growth arrest and DNA damage β (GADD45β), a NF-κB-regulated anti-apoptotic protein, able to suppress the JNK-mediated pro-apoptotic signaling by targeting MKK7 [93,94,95,96,97]. Recently GADD45β has been identified as an essential modulator of TAMs reprogramming and of the CD8^+^ T-cell trafficking in HCC tumors [98]. The authors demonstrated that *Gadd45b*^−/−^ mice displayed a reduced number of HCCs compared to WT mice. Indeed, *Gadd45b*^−/−^ HCC tumors, but not *Gadd45b*^+/+^ tumors, showed an enhanced F4/80^+^ and IBA1^+^ TAMs and T-cells infiltration as well as a higher number of tertiary lymphoid structures (TLSs), which are correlated to a favorable clinical outcome in most human cancers. Strikingly, M1-like macrophages were observed in *Gadd45b*-deficient HCC tumors, characterized by high expression of inflammatory markers, such as iNOS, COX-2 and MHC-II than *Gadd45b*^+/+^ HCCs, suggesting that Gadd45β loss increases the M1-like polarization state via upregulation of p38 signaling. These findings show that Gadd45β supports anti-inflammatory TAM activation and blocks TLS formation and lymphocyte infiltration within tumors, thus sustaining tumor growth [98]. However, in other tumors, the authors also demonstrated that macrophage-specific Gadd45β loss blocks oncogenesis, indicating that Gadd45β governs the immunosuppressive activity of the TME across multiple cancer types [98,99]. Therefore, targeting Gadd45β, in combination with conventional immunotherapies, could be a potential new therapeutic approach to counterstain Gadd45β-mediated tumorigenesis in human cancers. 

Receptor-interacting protein 140 (RIP140) is a protein widely expressed in macrophages, responsible for the regulation of TAMs energy metabolism and inflammatory response that has been associated with NF-κB pathway [100]. In HCC, NF-κB/IL-6 axis induces the alternative polarization of TAMs. In contrast, the overexpression of RIP140 in TAMs can reduce the expression of M2-like polarized markers (Arg-1, Peroxisome proliferator-activated receptors (PPAR), CD206) and enhance the expression of TNF-α, an M1-like marker. In vivo studies demonstrated that the inhibition of M2 polarization reduces tumor burden and invasiveness (proliferating cell nuclear antigen (PCNA) and VEGF ratio) and increases HCC cell apoptosis. In addition, they observed a decrease of p-p65, p-c-Jun and tumor necrosis factor receptor-associated factor 3 (TRAF3) expression levels, as well as of IL-6. These findings suggest that the overexpression of RIP140 inhibits NF-κB activation and influences TAMs polarization in HCC [100].

Contrastingly, Sharen et al. demonstrated that the infiltration of M1-TAM in HCCs induces a reduction of the efficacy of postoperative transcatheter arterial chemoembolization (TACE) in patients with liver cancer. In particular, the infiltration of M1 TAMs promotes the hyperactivation of NF-κB pathway, the increase in anti-apoptotic activity, the upregulation of the expression of cyclin-dependent kinase (CDK)1, CDK2 and cyclin D1, and the reduction of p21 expression in HCC cells, sustaining tumor proliferation. The pro-tumoral effect of M1-like TAMs was reverted by the administration of the p65 inhibitor, JSH-23, underlying the controversial role of NF-κB in M1 TAMs in HCC progression [101].

The use of immunotherapy for HCC treatment is still far from achieving its intended effect in the clinical setting, even if increasing number of patients have greatly benefited from this therapeutic approach [102,103,104,105,106,107].

The inhibitors of the programmed cell death protein-1 (PD-1)/programmed death-ligand 1 (PD-L1) pathway are one of the most used immune checkpoint inhibitors able to re-modulate T-cells and TAMs immune response [108]. Recently, Xu and collaborators demonstrated that the combination therapy using a Listeria monocytogenes-based tumor vaccine, Lmdd-MPFG, and anti-PD-1 reduced HCC tumor volume and increased survival rates, by inducing CD8 T-cells activation and IFN-γ signals. No effects are shown following monotherapy with anti-PD-1. In addition, they observed a shift of TAMs from M2 to M1 phenotype, an increased autophagy, driven by enhanced activation of the NF-κB pathway. This evidence highlighted that the combination therapy synergizes to affect TAM polarization and TME composition in a NF-κB dependent manner [109].

The liver is the preferred metastatic site for several tumors, such as colorectal cancer (CRC), lung cancer, melanoma, and gastric carcinoma. TAMs promote tumor progression by increasing tumor metastasis and stabilizing a pre-metastatic microenvironment [110,111]. Studies conducted by Li and collaborators demonstrated that N-myc downstream-regulated gene 2 (NDRG2), a protein that binds PTEN via protein phosphatase 2A (PP2A) recruitment, controls TME remodeling, and TAMs polarization during metastatic processes through activation of the NF-κB pathway. Interestingly, in a liver metastasis model, they found that Ndrg2^−/−^ macrophages have a tumor-suppressor phenotype compared to WT macrophages, and the M1–like polarization is driven by enhanced activation of the NF-κB pathway. Accordingly, the inhibition of IκBα phosphorylation abolishes the tumor-suppressor function of Ndrg2^−/−^ macrophages, thus inhibiting cancer liver metastasis [112].

### 3.2. Breast Cancer

The role of M2 TAMs in promoting proliferation, invasion, migration and angiogenesis in human breast cancer seems to be related to the NF-κB-mediated expression and secretion of galectin-3, a member of the β-galactoside binding proteins. Galectin-3 results particularly expressed in hypoxic tumor regions, where it promotes the migration and invasiveness of breast cancer cells and enhances angiogenesis and vascular mimicry. The expression of galectin-3 in TAMs is dramatically reduced in the presence of the NF-κB inhibitor, pyrrolidine dithiocarbamate (PDTC), both in normoxia and hypoxia conditions. In contrast, the presence of ROS induces the nuclear accumulation of NF-κB and the consequent upregulation of galectin-3 [113].

The chemokine profiling of murine and human breast cancer models indicated that CXCL1 is one of the most abundant chemokines secreted by TAMs. The levels of CXCL1 are significantly higher in metastatic lung cancer specimens compared to primary breast cancer tumor, suggesting the active role of CXCL1 in the promotion of breast cancer cell migration and invasiveness. Indeed, the presence of CXCL1 significantly increased breast cancer 4T1 cell migration and invasiveness ability, matrix metalloproteinase-2 (MMP-2) and MMP-9 secretion, as well as the expression of EMT-related proteins in vitro. TAMs-secreted CXCL1 mediates the EMT in breast cancer cells and induces the NF-κB-mediated expression of several metastatic genes, such as SRY-box transcription factor 4 (SOX4). ChIP assay showed that NF-κB actively binds the predicted binding region on the SOX4 promoter after CXCL1 treatment. Accordingly, the inhibition of NF-κB blocks CXCL1-induced SOX4 overexpression, increases the E-cadherin and reduces both vimentin and β-catenin expression, underlying the importance of CXCL1/SOX4/NF-κB axis in sustaining breast cancer [114]. 

Annexin 1 (ANXA1) is a protein member of the annexin family with multifunctional roles in cancer development and progression. ANXA1 is highly expressed in metastatic and triple negative (estrogen, progesterone and HER2 receptor) breast cancer (TNBC). Studies conducted on ANXA1^+/+^ and ANXA1^−/−^ mice indicate that ANXA1 is necessary for a macrophage phenotypic switch from M1 to M2. Interestingly, ANXA1 directly induces ERK and NF-κB activation via the formyl peptide receptors 2 (FPR2), leading to a macrophage polarization and tumor cell proliferation [115].

The physical structure of the tumoral extracellular matrix (tECM) in breast cancer has an important role in the protection of the tumor niche and in the penetrance of the immune system. TAMs redefine the composition of the tECM by enhancing the deposition of the heparan sulfate proteoglycan 2 (HSPG2), known as perlecan, that confers mechanical rigidity to the ECM and generates a favorable gradient for cancer cell development. NF-κB p65/p52 complex is responsible of the transcriptional regulation of HSPG2 in M2 TAMs and is associated with the altered expression of perlecan. Accordingly, the treatment with QNZ, the NF-κB inhibitor, on TAMs significantly downregulates the expression of HSPG2, indicating that NF-κB pathway is involved in the remodeling of the tECM and in the tumor escaping from the immune system [116]. 

The acquisition of properties associated with mammary stem cells (MaSCs) and cancer stem cells (CSCs) is one of the key passages that enables breast cancer cells to perform the epithelial mesenchymal transition (EMT) and develop malignancy, invasiveness and therapy resistance [117,118,119]. Interestingly, human monocytes or TAMs fail to promote tumor-initiation or progression in breast cancer cells expressing an NF-κB super-repressor, indicating that NF-κB activation and cytokines production have a critical role in the CSCs and in the tumorigenic effects of the TAMs [119].

Human mammary epithelial cells responsible for EMT were characterized as CD44^+^ and CD24^−^ [120]. Interestingly, mammary epithelial cells cocultured with M2 TAMs exhibit increased percentage of CD44^+^/CD24^−^ and elevated levels of HIF-1α, α-catenin, NF-κB, and twist family bHLH transcription factor 1 (Twist 1). In fact, the silencing of NF-κB in the M2-like MDA-MB-231 activated macrophages decreases the migration and invasion abilities of breast cancer cells. Mechanistically, NF-κB directly downregulates the anti-metastatic miR-488 and enhances special AT-rich sequence-binding protein-1 (SATB1) and Twist1 expression, the major initiators of the EMT, suggesting that the NF-κB/miR-488 axis is responsible for cancer cells migration and metastasis in presence of M2-TAMs [121]. 

It is known that the intercellular communication between TAMs, TME and several cancer cells, including breast cancer cells, is also mediated by EVs [122]. A recent study uncovered that TAM-derived EVs enriched with miR-660 inhibit Kelch-like protein 21 (KLHL21) in breast cancer cells leading to the activation of NF-κB p65 signaling pathway. In turn, the activation of NF-κB controls the cancer cells invasiveness, migration, and metastasis by modulating the expression of E-cadherin, N-cadherin, MMP-9, MMP-3 and β-actin [123].

### 3.3. Colon Cancer

Several pieces of evidence showed that TAMs sustain the initiation and progression of CRC by regulating several processes including tumor proliferation, metabolism, immunosuppression, angiogenesis and metastasis [124]. In CRC, intratumoral macrophages exert tumor-promoting functions, while TAMs found in the tumor invasive front correlate with better prognosis [122,123]. However, mixed macrophage populations are observed in human colorectal cancer tumors [125]. 

Studies in colitis-associated cancer (CAC) and in genetically driven Apc^Min^ mouse models showed that NF-κB pathway regulates the shift from M1 to M2 macrophages and the transition from colitis to colon cancer. While the accumulation of p50 induces the activation of the M2-like transcriptional program, p50 deficiency reduces inflammation and number and size of neoplastic lesions in chemically induced CAC. Similar results were found in genetically driven CRC, where tumor incidence and growth are reduced in Apc^Min^p50^−/−^ mice compared to Apc^Min^ mice. Accordingly, tumor inhibition was observed in the immunogenic MC38 transplantable CRC model in p50^−/−^ mice compared to WT mice, as well as an increased expression of M1 genes and cytokines. Moreover, p50 ablation reduces TAMs accumulation indicating the fundamental role of NF-κB in modulating macrophage infiltration [126]. Consistently, the inhibition of NF-κB (p50) in M2-TAMs by siRNAs induces M2 macrophages to switch towards M1-like phenotype, thus promoting the production of pro-inflammatory and tumoricidal factors, as well as growth factors and NO. These findings suggest that NF-κB plays a central role in the assessment and maintenance of TAMs phenotype in colon cancer [127]. 

Prolyl hydroxylases domain proteins (PHDs) are dioxygenases activated in response to oxygen availability. The overexpression of PHD2 isoform is associated with the suppression of colon cancer growth and invasiveness [128]. Interestingly, the reduced p65 phosphorylation and the downregulation of NF-κB-related downstream genes involved in cell cycle, EMT, and inflammation (e.g., CyclinD1, E-cadherin, and TNF-α) as well as in the recruitment of M2-like TAMs within the TME, has been found in colon cancer cells expressing PHD2 and in PHD2-overexpressing colon cancer xenografts, suggesting that NF-κB mediates the anti-inflammatory and anti-cancer effects of PHD2 in colon cancers [129]. 

Recent studies demonstrated that prolonged and enhanced NF-κB signaling activation sustains TAMs F4/80-positive recruitment and angiogenesis via P2X purinoceptor 7 (P2X7R) in CRC cells [130]. It is known that NF-κB sustains cancer cells survival and progression by regulating metabolic switch and adaptation to low nutrient conditions [33,131]. In vivo studies demonstrated that a ketogenic diet (KD) induces a strong p65 inhibition in both TAMs and CRC cells, that in turn, promotes TAMs M1 polarization and reduces tumor growth and metastasis, suggesting that NF-κB plays a role in the modulation of tumor metabolic adaptation and inflammatory response [132]. It has been demonstrated that metabolism is also associated to the TAMs ability to migrate with cancer cells to metastatic sites [133,134].

In CRC, macrophages have distinct metabolic profiles based on their migratory abilities. In fact, migration-active macrophages express lower abhydrolase domain containing 5 (ABHD5), a catalysator of triglyceride hydrolysis, compared to the nonmigratory TAMs. Interestingly, ABHD5 deficiency and, consequently, triglyceride accumulation, stimulate NLRP3 inflammasome activation, in a ROS-dependent manner, and induce IL-1β secretion, that in turn, stimulates p65 signaling. Conversely, gene microarray showed that ABHD5 suppresses NF-κB-dependent MMPs expression and activity, fundamental for cancer cell migration. In addition, the authors demonstrated that ABHD5-mediated knockdown (ABHD5-KD) macrophages have an enrichment of JAK–STAT and TLR pathways, that stimulate the NF-κB and JNK signaling compared to controls. In conclusion, TAMs expressing ABHD5 exert an inhibitory effect on colon cancer cell migration, while ABHD5^low^ macrophages promote NF-κB-dependent colon cancer cells invasiveness [135].

### 3.4. Glioblastoma

It is known that up to 30% of the total glioblastoma (GBM) composition is represented by peripheral macrophages and microglia [136,137]. In a GBM syngeneic immune competent mouse model, it has been shown that the LysM-Cre-mediated conditional deletion of p65 in myeloid lineage induces the TAMs polarization from the M2 to M1 phenotype. Indeed, p65 deficiency enhances CD8^+^T cells proliferation and increases levels of IFN-γ, TNF-α and IL-1β, which lead to a decreased GBM tumor growth. In contrast, where p65 KO and control donor mouse bone marrow was transplanted in immune-deficient mice, no reduction in tumor growth was observed in p65 KO chimaera mice compared to control mice. These findings suggest that the anti-tumor immunity in GBM is inextricably linked to T cells activity and is negatively regulated by the NF-κB pathway [138]. Interestingly, in a GL261-implanted mouse model, the treatment with curcumin (CC) induced an overall TAM repolarization from the M2 to the M1 phenotype via STAT1 and NF-κB signaling activation. Indeed, the CC treatment suppresses p50 homodimers in the microglia and promotes the activation of the p50/p65 NF-κB pathway, which leads to the M1 tumoricidal phenotype activation. Moreover, the concerted activation of STAT1 and NF-κB pathways induces apoptosis of GBM cancer cells and increases iNOS and Iba1^+^ expression in the microglia, indicating the presence of activated M1 macrophages which eliminate the remaining GBM cells and apoptotic debris, and a tumor remission in 50–60% of treated mice [139]. 

GBM growth is significantly accelerated by IL-1β, a cytokine highly expressed in TAMs and neutrophils localized in the perinecrotic tumor areas. IL-1β expression in TAMs is synergistically activated by CXCL8 and IL-6, which in turn activate NF-κB and STAT3 pathways. TAMs-derived IL-1β stimulates GBM tumor cells and induces the expression of IL-1β itself and CCL2, which actively recruits TAMs in the tumor site. Furthermore, damage-associated molecular patterns (DAMPs) released from necrotic cells stimulate IL-1β expression in TAMs with an autocrine/paracrine feedback loop that accelerates TAMs migration and tumor growth [140]. Consistently, higher IL-1β expression is correlated with a worse clinical course in GBM [33,131] suggesting that NF-κB /IL-1β axis in TAMs has a key role in GBM growth and progression.

### 3.5. Gynecologic Cancer

As for HCC, NF-κB/Gadd45β axis governs the immunosuppressive activity of the TME and the M2 macrophage polarization in ovarian cancer (OC) [98]. Therefore, the *Gadd45b* ablation in macrophages re-establishes proinflammatory TAM activation and CD8^+^ T-cell infiltration into the tumor, thus inhibiting ovarian adenocarcinoma growth [98]. 

The crosstalk between endothelial ovarian cancer cells (EOC) and TAMs promotes the progression of OC [141], and exosomes have been identified as principal mediators of this interplay [133]. In fact, exosomes isolated from TAMs in the ascites of EOC patients and carrying high levels of miR-146b-5p are then internalized in the cytoplasm of EOCs as endosome-like vesicles. Once internalized in EOCs, miR-146b-5p directly targets and inhibits the TRAF6/NF-κB/MMP2 axis, suppressing endothelial cell migration. In contrast, exosomes derived from EOCs, that transport 2 lncRNAs, restore the endothelial cell migration by activating NF-κB pathway in the recipient cells [142].

Mitochondrial transcription factor B2 (TFB2M), responsible for the mitochondrial DNA (mtDNA) transcription and compacting, is expressed in ovarian cancer patients, and is associated with poor prognosis, immunosuppressive TME and macrophage polarization. In fact, IHC analysis on primary OC specimens indicates a striking correlation between TFB2M expression and M2 TAM infiltration. Interestingly, the overexpression of TFB2M increases cytosolic mtDNA and IL-6 expression in OC cells via TLR9/NF-κB signaling pathway activation, contributing to M2 macrophage polarization and infiltration in the TME [134]. Accordingly, the inhibition of cytosolic mtDNA, TLR9 or NF-κB pathway blocks the TFB2M-induced IL-6 expression and abrogates the M2 macrophage polarization and the immunosuppressive TME in OC. 

In other types of gynecologic cancers, TLR9 pathway activates lymphocyte T cell shift towards Th1 profile and decreases the number of MDSCs, TAMs and Tregs in the TME, displaying anti-tumor properties [133]. However, the TLR9 pathway stimulated by radiotherapy, cell death, and CpG release, can also induce tumor progression, angiogenesis and invasiveness in cervical cancers through the activation of the NF-κB and STAT3 transcription factors [143].

As extensively reported, hypoxia plays a fundamental role in cancer progression, and hypoxic regions are often infiltrated by immunosuppressive cells, such as TAMs, MDSCs and Tregs [144,145] and are strongly associated with poor patient outcomes [146]. It has been demonstrated that hypoxia induces the binding of HIF-1α to the proximal promoter of zinc finger E-box binding homeobox 1 (ZEB1), which is highly expressed in cervical cancer hypoxic cells islets and is associated with a stronger pro-tumor phenotype. ZEB1-driven cancer cells produce CCL8, attract and promote macrophages activity through NF-κB activation. Interestingly, TCGA data showed that both ZEB1 and CCL8 overexpression correlate with poor prognosis in human cervical cancer. These findings suggest that the NF-κB pathway is responsible for hypoxia-induced ZEB1-mediated TAM infiltration, and targeting ZEB1-CCL8-NF-κB axis could be a promising strategy for the cervical cancer treatment [147]. 

### 3.6. Other Solid Tumors

Other solid tumors in which TAMs have a crucial role in mediating tumor progression and immune escape are neuroblastoma (NB), bladder cancer, renal carcinoma, gastric cancer (GC), basal cell carcinoma (BCC), oral squamous cell carcinoma (OSCC), prostate cancer (PCa), nasopharyngeal carcinoma (NPC), multiple myeloma (MM), sarcoma, pancreatic cancer (PDA) and lung cancer [148,149,150,151,152,153,154,155,156,157,158,159,160,161,162,163]. In addition, it is known that in these tumors TAMs correlate with worse prognosis and drug resistance [164]. 

NB is the most frequent pediatric cancer, with heterogeneous clinical outcomes based on tumor stage [165]. Recently, it has been demonstrated that higher levels of lipid metabolism-related genes are associated with unfavorable histology and an advanced stage of NB compared to the early stage. Fatty acid binding protein 4 (FABP4), a lipid chaperone that facilitates lipid distribution and response in cells, is highly expressed in NB macrophages compared to other cell types of the TME, leading to tumor progression [137]. In NB orthotopic and metastatic mouse models with FABP4 KO macrophages the tumor growth is repressed. Accordingly, the authors observed an increased tumor growth when FABP4 is overexpressed in macrophages. Furthermore, FABP4 inhibits NF-κB activity and IL-1α secretion in TAMs and downregulates ATP production via the ubiquitination of ATPB, maintaining TAMs in an anti-inflammatory state and sustaining the pro-tumorigenic effects on NB cells [137]. 

Bladder cancer is the fourth most common cancer in men and the overall survival is still low [149]. In vitro studies with HTB-1 and T24 bladder cancer cell lines cocultured with M2-like TAMs, demonstrated that the coculture upregulates the expression of EMT-related genes, such as VEGF, twist, vimentin and NF-κB in bladder cancer cells, leading to increased migratory and tumor sphere generation abilities. Interestingly, the treatment with BAY11-7082, a potent NF-κB signaling inhibitor, reduces the expression of the CD206, a M2-macrophage marker, and increases the expression of TNF-α, a M1 marker. In addition, the treatment reverts the EMT-induced phenotype of bladder cancer cells and reduces the expression of CD133^+^, a marker produced by M2-like TAMs. The authors also showed that BAY11-7082 induces the expression of miR-30a both in bladder cancer cells and in TAMs, reprogramming M2-like macrophages towards M1 phenotype. These findings underline the role of NF-κB in the regulation of TME and cancer cells metastatic potential in bladder cancer [166]. 

In human renal cell carcinoma (RCC), the most common type of kidney cancer in humans [167], blood derived monocytes display a distinct transcriptional profile characterized by the upregulation of pro-inflammatory cytokines and chemokines, pro-tumor genes, (e.g., COX2, IL-8, VEGFA, MMP19, MMP10, CXCR4, and HIF1A) and polarization-related genes [168]. Interestingly, RCC-conditioned monocytes display enhanced IκBα phosphorylation and p65 NF-κB nuclear translocation, via IL-1/IL-1R/MyD88 signaling activation. In RCC mouse models, the blockade of IL-1/IL-1R pathway with recombinant IL-1RA or Il1r1 knockdown, inhibits tumor growth and TAMs pro-tumor phenotype. Meta-analysis of tumor gene-expression data from RCC patients highlights that the higher expression of IL-1B correlates with pro-tumor genes induced by TAMs and advanced tumor stages, indicating that IL-1/IL-1R signaling is crucial in shaping the TME and the RCC tumor development through MyD88-dependent NF-κB pathway activation [169]. 

GC is the fifth leading cause of death worldwide [158]. Immunostaining of primary GC tissue samples suggests that TAMs are preferentially localized in the invasive tumor front and correlate with invasion, lymph node metastasis, TNM stage, and poor prognosis [170]. In vitro co-culture of GC cells and macrophages indicates that TAMs actively produce angiogenic and lymphangiogenic growth factors, VEGF, and VEGF-C, through the activation of NF-κB signaling pathway. Interestingly, NF-κB inhibition in TAMs decreases the expression of VEGF and VEGF-C in both macrophages and cancer cells, but the molecular mechanism is still unclear. However, this study indicates that NF-κB has a role in the interactions between tumor cells and TAMs in GC [170].

BCC is the most common skin cancer with a complicated pathogenesis [160]. In aggressive (micronodular) BCC, increased tumor invasion and angiogenesis correlates with the number of TAMs [171]. Using an in vitro noncontact co-culture system, Tjiu and colleagues demonstrated that both M2-polarized THP-1 and monocyte-derived M2 macrophages promote extracellular matrix degradation, invasiveness and angiogenesis of BCC cells by inducing COX2-dependent MMP-9, VEGF-A and basic fibroblast growth factor (bFGF) expression, via the p38 MAPK/NF-κB cascade [171]. 

Another skin cancer with a considerable tumor mutational burden is melanoma. Melanoma is the most fatal skin cancer and TAMs play an important role in regulating tumor development [161]. In situ immunofluorescence analysis of human primary melanoma samples indicates that there is a strong correlation between the abundance of TAMs expressing CCL20/TNF/VEGF-A and worse prognosis. The study illustrates that this pro-tumoral M2 phenotype of TAMs is driven by a consistent p53 and NF-κB activation [172]. 

TAMs promote tumor progression also in OSCC, a common head and neck tumor with a low overall survival [162]. It is known that the TAMs phenotype is related to Axl signaling, an oncogenic pathway activated by the autocrine and paracrine release of growth arrest—specific 6 (Gas6), an antiapoptotic and proliferative protein [173,174]. In vitro studies showed that OSCC cells expressing Axl polarize THP-1 towards M2 phenotype and induce the secretion of pro-tumorigenic factors like MMP2, MMP9 and VEGF, by activating the PI3/Akt/NF-κB pathway. Accordingly, the inhibition of Axl, PI3/Akt and NF-κB on OSCC cells with specific single inhibitors, reduces M2 polarization [175]. Furthermore, co-culture of OSCC cells with THP-1 cells enhances the expression of Axl and its ligand, Gas6, and NF-κB transcription activity in cancer cells that, in turn, acquire tumor invasion/migration abilities and express EMT related genes [176]. These findings suggest the presence of a bidirectional interaction between TAMs and OSCC that promotes malignancy and tumor growth via Gas6/Axl/NF-κB signaling [175,176].

The expression of nephroblastoma overexpressed (NOV)/ cellular communication network factor 3 (CCN3) has been associated with M2 macrophage infiltration in PCa. PCa is the second most common tumor in men. NF-κB plays an important role during prostate cancer progression and its overexpression is correlated with worse prognosis [163,177]. CCN3 secreted by PCa cells recruits macrophages and educates TAMs toward an M2 phenotype, while PCa cells pre-treated with CCN3-neutralizing antibody attenuate CCN3-induced macrophage migration and polarization. Interestingly, in RAW264.7 cells, CCN3 induces the phosphorylation of focal adhesion kinase (FAK), Akt, and NF-κB, in a time-dependent manner, and enhances p65 binding to the NF-κB element on the VEGF promoter, leading to increased angiogenesis. Correspondingly, pre-treatment with FAK, Akt, and NF-κB inhibitors, or transfection with a dominant-negative (DN) mutant of FAK, Akt, IKKα, or IKKβ, abolishes the VEGF expression. Consistently, knockdown of CCN3 in PCa cells inhibits RAW264.7-promoted angiogenesis and tumor growth in mouse models, suggesting that CCN3 secreted by PCa cells regulates TAMs infiltration and function by modulating the FAK/Akt/NF-κB signaling pathway in macrophages [178].

MM cells can modify the bone marrow (BM) niche and induce M2 phenotype polarization by releasing several soluble factors [179]. A recent study showed that circulating miR-16 in MM patients is associated with a better survival, and MM patients with chromosome 13 deletion (Del13) express lower levels of miR-16 compared to non-Del13 patients. Interestingly, primary basal state spleen MΦ (M0-MΦ) isolated from miR-16 KO mouse showed a more pronounced M2 phenotype compared to WT mice. The treatment of MΦ isolated from MM patients and mice, MΦ-like malignant cell line and human stromal cell line with ds-miR-16 significantly downmodulates the expression of both IKKα and IKKβ. Consistently, miR-16 directly binds IKKβ 3′UTR and subsequently affects protein translation. Indeed, miR-16 has a significant additive effect in inhibiting NF-κB activity in MM cells when combined with bortezomib or BAY11-7082 and sensitizes MM-PCs and resident MΦ of the BM-ME to bortezomib treatment. These findings indicate that miR-16 mediated-NF-κB pathway activation has a critical role in the selection and polarization of specific monocytes clones during MM progression [180].

The development and progression of nasopharyngeal carcinoma (NPC) is associated with Epstein–Barr virus (EBV) infection and sustained inflammation, due to the inhibition of forkhead box P1 (FOXP1) tumor-suppressive activity driven by one of the 44 EBV miRNAs, EBV-miR-BART11. In fact, EBV-miR-BART11 has been found to be significantly higher in NPC biopsies compared to non-tumor nasopharyngeal epithelial tissues. Inhibition of FOXP1 induces enhanced NPC cells proliferation and the expression of inflammatory factors (e.g., IL-1β, IL-6, and IL-8) leading to a sustained local inflammation. Moreover, macrophages transfected with EBV-miR-BART11 are hyperresponsive to LPS and promote epithelial cell proliferation. Indeed, FOXP1 overexpression decreases NF-κB p65 protein expression, while EBV-miR-BART11 overexpression or FOXP1 knockdown increases NF-κB p65 expression, indicating the role of NF-κB in the crosstalk between NPC cell and TAMs in response to inflammation and tumor development [181].

In Ewing sarcoma (ES), the regulation of macrophages infiltration is driven by let-7a expression. The let-7a has a tumor suppressive activity and inhibits TAMs recruitment on tumor site. The overexpression of let-7a represses STAT3 signaling pathway, which is constitutively activated in ES cells. Accordingly, STAT3 upregulation suppresses let-7a activity by inducing NF-κB pathway, that inhibits the expression of mature let-7a through lin-28B, leading to enhanced TAMs infiltration and activation [182]. 

It has been demonstrated that acidic polysaccharide IAPS-2 exhibits an anti-tumor effect on sarcoma by re-educating TAMs towards an anti-tumor phenotype. The IAPS-2 treatment induces NF-κB and STAT1 pathways and inhibits STAT3 signaling, that in turn, modulates TAM gene expression and cytokine profile. In S180 tumor-bearing mice, IAPS-2 promotes the secretion of several M1 markers and reduces the concentration of MMP-9 and VEGF in the tumor, restoring the immunosurveillance with an anti-angiogenetic effect [183]. 

In pancreatic (PDA) cancer, the transcription factor Cut like homeobox 1 (CUX1) is an important modulator of TAM phenotype plasticity and functions and it is highly expressed not only in PDA tumor cells but also in PDA TAMs. It is recognized that CUX1 displaces NF-κB p65 binding at the promoter level of CXCL10 and recruits HDAC1, which decreases the acetylation and transcriptional activity of NF-κB, leading to a reduced expression of M1 phenotype-related cytokines. As a result, CUX1 reduces the recruitment and the activation of M1 TAMs, promoting tumor progression and angiogenesis in PDA. These finding suggest that CUX1 regulates TAMs phenotype by counteracting NF-κB activity [184]. It is established that ApoE mediates cholesterol metabolism, is highly expressed by TAMs and CAFs both in mouse and human PDA and correlates with patient survival. Recently, Kemp and colleagues showed that apolipoprotein E (ApoE) sustains immunosuppressive TME in PDA via NF-κB-mediated CXCL1/5. Specifically, ApoE expressed in tumor macrophages binds LDLR and induces CXCL1/5 expression through NF-κB signaling [185]. Accumulating evidence showed that lung cancer displays alveolar macrophages (AM), the tissue-resident macrophages, which exhibit different functions within the TME. As for other cancers, TAMs sustain proliferation, immunosuppression and invasion in lung cancer via secretion of several cytokines, chemokines and growth factors [155]. Analysis of non-small cell lung cancer (NSCLC) tumors from 60 patients indicate that plasminogen activator inhibitor-1 (PAI-1) expression correlates with TGF-β expression and the percentage of TAMs. In vitro study has demonstrated that PAI-1 on cancer cells binds TLR4 and promotes TGF-β secretion in macrophages by increasing IL-6 production via NF-κB activation. At the same time, TGF-β promotes PAI-1 expression in NSCLC cells in a R1/SMAD3-dependent manner. However, while a high concentration of TGF-β can inhibit NF-κB activation, optimal concentration of TGF-β activates the NF-κB pathway and IL-6-induced TGF-β production. These findings indicate the presence of an auto-regulatory or auto-inhibitory loop regulated by NF-κB activation responsible for the immunosuppressive TME in NSCLC [186].

## 4. Targeting NF-κB Pathway in TAMs

It is recognized that TAMs are involved in orchestrating the immune response, tumor growth and progression as well as metastasis and TME remodeling. In particular, the polarization of TAMs to the M1 phenotype exerts important tumor suppressing effects on the TME, while the M2-like macrophages display pro-tumoral and angiogenetic activity [11]. NF-κB plays a key role in the regulation of TAMs as it swings the scale between tumor suppression and tumor progression [15]. Thus, there is a rationale to target NF-κB pathway to counteract TAMs activity within the TME. In this contest, the principal strategies explored are (a) re-education of TAMs towards the antitumoral (M1) phenotype; (b) TAMs depletion; (c) termination/blocking of macrophage recruitment and accumulation into the TME (Table 2). Although all these strategies showed efficacy in early-stage diseases, recent evidence suggests that the TAM reprogramming approach could be safer and more effective.

### 4.1. Re-Educating TAMs via NF-κB Modulation

A promising and highly investigated approach engages toll like receptors (TLRs) to induce an immunostimulatory response and reshape TAMs into M1 phenotype. Several studies using bioactive compounds such as chrysin thiazole derivative (ChR-TD), anemoside A3 (A3), β-D-(1→6) glucan (AAMP-A70), aqueous extract of Cimicifuga dahurica (CRAE) as well as antineoplastic agent (e.g., paclitaxel) demonstrated that these drugs activate TLR2 or TLR4 that in turn directly trigger the NF-κB pathway leading to TAMs reprogramming toward M1-like antitumor phenotype. In vitro studies demonstrated that these compounds induce a significant increase of TNF-α, IL-12, IL-6 and IL-1β mRNA expression and downregulate M2-like genes like IL-10, Ym-1, CD206 and Arg-1 in several models of cancer (e.g., breast cancer, colon cancer, MM) [187,188,189,190,207]. Accordingly, homogeneous polyporus polysaccharide (HPP) induces M1 polarization via TLR2/NF-κB/NLRP3 signaling activation and reduces the progression of bladder cancer [191].

The PI3K/NF-κB axis is another molecular target investigated for its capability to promote M1 polarization. He and colleagues demonstrated that the inhibition of PI3Kγ with the natural compound baicalein potentiates the M1 macrophage polarization and inhibits tumor growth via NF-κB/TNF-α inflammatory signaling in both breast cancer and melanoma mouse [57]. 

Hoover and collaborators showed that increased IKK2 activity, an enzyme involved in the NF-κB signaling pathway, in macrophages contributes to M1 TAMs polarization and abrogates ovarian cancer growth in vivo [192]. 

It has been shown that the use of BAY11-7082, a selected inhibitor of the NF-κB pathway, inhibits anti-inflammatory macrophage phenotype and reduces the invasiveness of bladder cancer cells by increasing the expression of miR30a [166]. Additionally, targeting IncRNA DCST1-AS1/NF-κB axis in oral epithelial squamous cell carcinoma (OSCC) blocks the M2 polarization and tumor progression [193]. 

Recent and innovative approaches have exploited nanoparticle technology to restrain TAM activity. An interesting attempt has been made with glycocalyx-mimicking nanoparticles (GNPs), both in vitro and in vivo. These nanoparticles are specifically internalized by TAMs and can neutralize and reprogram M2 TAMs through the reciprocal STAT6 suppression and NF-κB phosphorylation. In addition, the authors demonstrated that GNPs improve the therapeutic efficacy of anti-PDL1 and reduce Lewis lung carcinoma (LLC) growth [194]. Congruently, mannose modified lipid nanoparticles (M-IMD-LNP), containing the NF-κB inhibitor IMD-0354, as well as hyaluronic acid (HA) nanoparticles, loaded with the micro-RNA miR-125, showed that M2 TAMs are able to repolarize toward the M1 phenotype, in an NF-κB dependent manner, both in melanoma cells and in an in vivo model of non-small cell lung cancer (NSCLC) [195,196]. Furthermore, Li and collaborators showed that a modified porous hollow iron oxide nanoparticles (PHNPs) loaded with a P13Kγ small molecule inhibitor (3-methyladenine, 3-MA), (PHNPs@DPA-S-S BSA-MA@3-MA), selectively inhibit the PI3Kγ/Akt signaling in TAMs by inducing a prolonged activation of NF-κB, through the reduction of the P13Kγ protein in macrophages and cancer cells, that in turn, promote the switch of TAMs toward pro-inflammatory M1 phenotype and the activation of immune response leading to reduced tumor growth and immunosuppressive TME [197]. 

The use of cell membrane-coated nanocarrier system such as PLGA-ION-R837@M, is another strategy to repolarize macrophages toward M1 and mitigate immunosuppressive TME by activating interferon regulatory factor 5 (IRF5) and NF-κB pathway using the synergy of Fe_3_O_4_ NPs and R837, a TLR7 antagonist [198]. As for PLGA-ION-R837@M, Gd@C82 nanoparticles modified with b-alanines (GF-Ala) showed an anticancer effect in vivo by activating the anti-tumor immune response and relieving the immunosuppressive TME via NF-κB/IRF5 activation [199]. 

In addition, the reprogramming of M2 macrophage in M1 via NF-κB pathway activation and the consequently tumor inhibition, was also observed in melanoma-bearing mice using copper sulfide nanoparticles (CuS-NP) [200].

Recently, Zhao and collaborators demonstrated that cetuximab exerts its antitumoral effect in colon cancer attenuating the M2 TAMs pro-tumorigenic activity and triggering the repolarization of TAMs from the M2 to the M1 phenotype. Cetuximab inhibits EGFR signaling and suppresses IL-6 expression in M2 TAMs by directly inhibiting the NF-κB and STAT3 pathways. Cetuximab-induced TAM repolarization strongly affects the immunosuppressive TME by reducing the tumor burden and inflammation in both the xenograft and azoxymethane/dextran sodium sulfate (AOM/DSS)-induced mouse colon cancer model [201]. Another drug able to re-programming TAMs toward a M1 phenotype is cabazitaxel. The RNA profiling of bone marrow–derived macrophage (BMDM) treated with cabazitaxel displays a significant TLR/NF-κB signaling activation and consequently the upregulation of NF-κB-mediated cytokines and chemokines expression compared to control. Indeed, rather than exerting a direct cytotoxic effect on breast cancer cells, cabazitaxel appears to induce an NF-κB-mediated macrophage’s polarization toward a M1 state stimulating the programmed cell removal (PrCR) macrophage activity [202]. 

According to the TAM re-polarization strategies, the treatment with proton irradiation has shown to be effective in the reprogramming of TAMs via modulation of NF-κB signaling. Proton therapy applied on THP1-derived M0, M1 and M2 phenotype macrophages promotes an enhanced nuclear translocation of NF-κB p65 in all three groups after 2h of irradiation. Both gene expression and cytokines analysis showed that moderate doses of proton irradiation promote the reprogramming of M0 and M2 macrophages towards an M1 phenotype. However, the combined treatment of irradiation with the NF-κB inhibitor Bay 11-7082 (IKK inhibitor) produces a clear induction of the macrophages towards the M2 phenotype. In particular, the M0 macrophages shifted to the M2 phenotype rather than M1 polarization, while a reinforcement of the M2 phenotype was observed in M2 macrophages [80].

### 4.2. TAMs Depletion and Termination of Recruitment via NF-κB Modulation

Recently, it has been demonstrated that CCL2, a pro-inflammatory and NF-κB-mediated chemokine, promotes TAM infiltration. In addition, NF-κB/CCL2 signaling pathway plays a key role in tumor progression, invasion and metastasis in a wide range of human cancers [203]. Therapeutic inhibition of CCL2/CCR2 axis through NF-κB ablation, abrogates inflammatory monocyte recruitment and TAM infiltration in different mice models of several cancer types such as primary and metastatic breast cancer, HCC and lung cancer [204,208]. In this contest, the natural compound total glucosides of paeony (TGP) downregulates the expression of genes involved in the formation and function of TAMs via NF-κB/CCL2 ablation as well as the secretion of CCL2 in primary and metastatic breast cancer. Furthermore, it decreases tumor growth and mitigates the immunosuppressive TME by decreasing CD45^+^CD11b^+^F4/80^+^ TAMs population and promoting CD4^+^ and CD8^+^ T cell infiltration in vivo [204].

The knocking down of IκBα protein obtained through the transfection of the IκBα si-RNA encapsulated into mannosylated siRNA-delivering NPs, promotes the recruitment of T-cells within the TME and inhibits macrophage infiltration both in ovarian and in breast cancer [205,209,210].

All trans-retinoic acid (ATRA) is a clinically available molecule able to immune-modulate myeloid cells. In a study on prostate cancer, it was shown that the treatment of primary macrophages with medium conditioned with PC3 prostate cancer cells induces a clear activation of the NF-κB and ERK pathways, and M2 macrophages polarization. Exposure to ATRA suppresses the production of the M2-like released factors (e.g., IL-10, IL-1β, indoleamine-pyrrole 2,3-dioxygenase (IDO) and VEGF) and downregulates MHC class I and II and Fas ligand (FasL) molecules expression, through a specific inhibition of NF-κB p50 without effects on ERK phosphorylation [206].

### 4.3. Targeting TAMs in Clinical Setting

It is known that NF-κB transcription factors are major drivers of most human cancers and yet there are no clinically useful NF-κB inhibitors to treat most of them, given the on-target toxicities of IKK/NF-κB targeting drugs. Currently, numerous cancer-selective agents targeting upstream activators or downstream effectors of the NF-κB pathway that are able to circumvent the adverse effects associated with the systemic targeting of NF-κB, are being tested in clinical studies [40,41,211,212,213,214,215]. However, none of these agents showed the capability to specifically target NF-κB in TAMs. Unlike NF-κB inhibitors, several other agents in clinical trials were proven effective in polarizing TAMs toward an anti-tumor phenotype when used alone or in combination therapy [216,217]. The use of TLR agonists, either as monotherapy or more commonly in combination with targeted therapy or immunotherapy, promotes TAMs-specific antitumor activity in advanced and refractory solid tumors [218,219]. Additionally, several agents targeting CSF1(R), CCR5, CD40, CD47 as well as CCL2-CCR2 have shown efficacy both as a single agent or in combination in patients with solid tumors [216,220,221,222,223,224,225,226,227]. Yet, recent evidence arising from ongoing clinical trials indicates that the functional reprogramming of TAMs could be a promising therapeutic approach to ameliorate the outcomes of cancer patients.

## 5. Conclusions

TAMs are the principal component of the TME, and infiltrating macrophages are associated with worse clinical outcome and drug resistance. Although numerous progresses have been made for cancer treatment, targeting macrophages represent a new approach in cancer immunotherapy to improve tumor immune microenvironment. NF-κB is a master regulator of innate immune responses and plays a key role in TAM reprogramming within the TME. However, while a great bulk of evidence has been collected, there are still controversies about the functions of NF-κB in myeloid cells within the TME. Hence, NF-κB activation in TAMs has been associated to the acquisition of both immunosuppressive and immunopermissive TAM phenotype, with opposite effects in terms of tumor progression. Therefore, further studies are needed to better understand in which tumoral contexts the inhibition of NF-κB signaling to reprogram TAMs could be of benefit.

## Figures and Tables

**Table 2:** Molecules/drugs targeting NF-κB pathways to reshape tumor microenvironment via termination of macrophage recruitment, TAMs depletion, and TAMs repolarization.

TAM REPROGRAMMING			
Molecules/Drug	Target	Cancers	Ref.
Chrysin thiazole derivative (ChR-TD)	TLR4/NF-κB	Breast cancer cell line (4T1)	[187]
Anemoside A3 (A3)	TLR4/NF-κB	Breast cancer	[188]
β-D-(1→6) glucan (AAMP-A70)	TLR2/Akt/NF-κB	Colon cancer cells	[189]
Aqueous Extract of Cimicifuga dahurica (CRAE)	TLR4/MyD88/TAK1/NF-κB	MM	[190]
Homogeneous Polyporus Polysaccharide (HPP)	TLR2/NF-κB/NLRP3	Bladder cancer	[191]
Baicalein	PI3K/NF-κB	Breast cancer, melanoma	[57]
IKK2	NF-κB	OC	[192]
BAY11-7082	NF-κB/miR30a	Bladder cancer cells	[166]
IncRNA DCST1-AS1	NF-κB	OSCC	[193]
Glycocalyx-mimicking nanoparticles (GNPs)	STAT6 and NF-κB	LLC	[194]
Mannose modified lipid nanoparticles (M-IMD-LNP) with IMD-0354	NF-κB	Melanoma cells (B16)	[195]
Hyaluronic acid (HA) nanoparticles, loaded with micro-RNA miR-125	NF-κB	NSCLC	[196]
Porous hollow iron oxide nanoparticles (PHNPs) loaded with 3-methyladenine (3-MA)	PI3Kγ/Akt/NF-κB	Breast cancer cell line (MDA-MB-231)	[197]
PLGA-ION-R837@M	TLR7/IRF5/NF-κB	Breast cancer cell line (4T1)	[198]
Gd@C82 nanoparticles modified with b-alanines (GF-Ala)	NF-κB/IRF5	Breast cancer cell line (4T1)	[199]
Copper sulfide nanoparticles (CuS-NP)	NF-κB	Melanoma	[200]
Cetuximab	NF-κB and STAT3	CRC	[201]
Cabazitaxel	TLR/NF-κB	Breast cancer cells	[202]
Proton irradiation	NF-κB	THP1 cells	[80]
**TAM DEPLETION AND TERMINATION OF RECRUITMENT**			
CCR2 antagonist or knocking out of host CCR2	CCL2/CCR2/NF-κB	HCC	[203]
Total glucosides of paeony (TGP)	NF-κB/CCL2	Breast cancer	[204]
IκBα si-RNA encapsulated into mannosylated siRNA-delivering NPs	NF-κB	OC, breast cancer	[205]
Trans-retinoic acid (ATRA)	NF-κB	PCa	[206]
